# Molecular dynamics simulation of the nanosecond pulsed electric field effect on kinesin nanomotor

**DOI:** 10.1038/s41598-019-56052-3

**Published:** 2019-12-23

**Authors:** Jiří Průša, Michal Cifra

**Affiliations:** 1 Institute of Photonics and Electronics of the Czech Academy of Sciences, Chaberska 1014/57, Prague, 18251 Czech Republic; 2 Faculty of Chemical Engineering, University of Chemistry and Technology Prague, Technicka 5, Prague, 16628 Czech Republic

**Keywords:** Protein analysis, Biophysical chemistry, Electrical and electronic engineering, Bionanoelectronics, Biological physics

## Abstract

Kinesin is a biological molecular nanomotor which converts chemical energy into mechanical work. To fulfill various nanotechnological tasks in engineered environments, the function of biological molecular motors can be altered by artificial chemical modifications. The drawback of this approach is the necessity of designing and creating a new motor construct for every new task. We propose that intense nanosecond-scale pulsed electric field could modify the function of nanomotors. To explore this hypothesis, we performed molecular dynamics simulation of a kinesin motor domain docked on a subunit of its microtubule track - a single tubulin heterodimer. In the simulation, we exposed the kinesin motor domain to intense (100 MV/m) electric field up to 30 ns. We found that both the magnitude and angle of the kinesin dipole moment are affected. Furthermore, we found that the electric field affects contact surface area between kinesin and tubulin, the structure and dynamics of the functionally important kinesin segments, including microtubule binding motifs as well as nucleotide hydrolysis site which power the nanomotor. These findings indicate that external intense nanosecond-scale electric field could alter kinesin behavior. Our results contribute to developing novel electromagnetic methods for modulating the function of biomolecular matter at the nanoscale.

## Introduction

**Figure 1:**
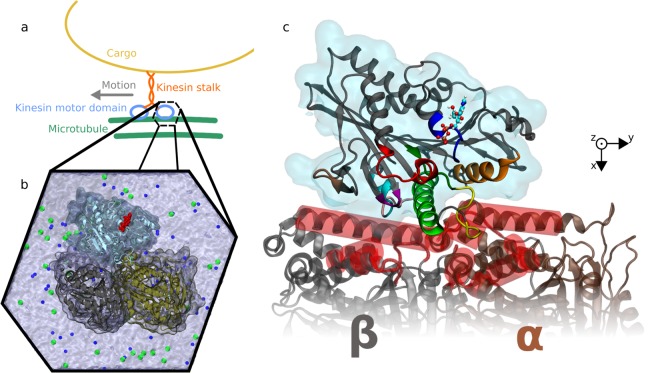
Nanomotor system analyzed in our molecular dynamics simulations: (**a**,**b**) kinesin motor domain docked on a tubulin heterodimer - subunit of the microtubule track. (**b**) the full all-atom molecular model used in simulations with water molecules and ions: tubulin heterodimer with kinesin motor domain (cyan), adenosine diphosphate (red), potassium ions K^+^ (blue) and chloride ions Cl^−^ (green) as dots. (**c**) close-up on kinesin motor domain on *α*, *β*-tubulin heterodimer: the *α* carbons in a whole tubulin except the red segments are restrained from motion to simulate the rigidity and mass of the whole microtubule. Important kinesin segments are color coded: nucleotide binding pocket containing P-loop (blue), switch I (red), switch II (dark green), and microtubule-binding motifs: loop L7 (purple), loop L8 (brown), loop L11 (yellow), *α*4 helix (light green), loop L12 (pink), *α*5 helix (cyan), and *α*6 helix (orange).

The use of nanomotors in engineered environments for nanotechnological purposes has gained significant attention over the last years^1^. The two main approaches to this topic are the development and use of synthetic machines, or the utilization of biological nanomotors. The clear main advantage of the latter approach is the fact that we can take the advantage of the biological design that has been fine-tuned by billions of years of evolution for the maximum efficiency, which in some cases is close to 100%^2^. Harnessing these nature-made nanomachines to perform nano-technological tasks, ranging from cargo shuttling to parallel computation, is in the center of a rapidly developing research field^3–6^. One of the best understood biological nanomotors is the motor protein kinesin-1^7^ (further referred to as kinesin). The kinesin consists of two identical globular catalytic domains termed the motor domains, connected by a long and thin stalk region, which can attach to a microscopic cargo (Fig. 1a). The two motor domains alternate to take 8 nm long steps along microtubules, bio-polymeric polar fibers, which serve as the track for this nanomotor, consuming chemical energy in the form of one molecule of adenosine triphosphate (ATP) per step. Kinesin steps always in one direction determined by the polarity of the track (towards the plus end - the *β*-tubulin end - of microtubule) without sidestepping^8,9^, taking several hundreds of steps before detaching from the track (property termed processivity) enabling thus long-range cargo transport. The maximum force that this nanomotor is capable of exerting is about 6 pN^10^.

To harness biological nanomotors, like kinesin, for nanotechnological tasks, it is essential to be able to modify their natural functional properties, such as directionality, side-stepping, processivity or force generation, to fit the particular task. One way of altering the nanomotor function, which has been explored during the recent years, are chemical modifications altering the nanomotor structure^11–13^. An obvious drawback of this strategy is that new species of the motor have to be designed and produced for each new task. An attractive alternative to this strategy is the creation of “switchable” motors, whose function can be altered by a noncontact modulation, such as a chemical^14^, light^15^ or electric signal.

Kinesin was termed as an electrostatic machine^16^ since electrostatic interactions bias binding to its track^17^ and their imbalance also plays role in kinesin dysfunction^18^. Therefore, we propose that intense (>MV/m) electric pulses of ultra-short (<100 ns) duration could modify the kinesin behavior on its track to modify its function. A way to predict if electric field (EF) is able to affect kinesin at this time scale and in what manner, is to perform molecular dynamics simulation where electric field is applied in the system^19^. In this way, the response of other protein structures to EF has been already probed^20–23^. It has been demonstrated that EF can affect conformation of pancreatic trypsin inhibitor^24^, insulin^25–27^, lysozyme^28–31^, *β*-amyloid and amyloid forming peptides^32,33^, and soybean hydrophobic protein^34^. Further, EF also caused myoglobin unfolding^35,36^, induced a *β*-sheet to a -helix-like conformation transition of peptides^37^, and caused structural destabilization of chignolin^38,39^ in molecular dynamics simulations. Moreover, recent molecular simulation studies demonstrated that EF can affect water diffusivity and ion transport across transmembrane proteins such as aquaporins^40–43^. There also recent experimental works which demonstrated that the protein conformation can be affected by intense nanosecond scale electric field^44,45^. While it is acknowledged that kinesin is an electrostatic nanomachine^16^, there is no molecular dynamics simulations work investigating the effect of external EF on kinesin nanomotor so far. To fill in this gap, we focus in this paper on how EF affects kinesin motor domain docked on a tubulin in terms of kinesin dipole properties, structure, and dynamics.

## Results and Discussion

### Kinesin structure selection

The optimized structures of kinesin motor domain (further referred to as “kinesin” for brevity) docked on tubulin heterodimer was obtained from Chakraborty^46^. The structures were available at the three states of nucleotide (ATP-like, ADP, APO). First, we formulated the rationale for the selection of the most appropriate nucleotide state. The rationale was to select the nucleotide state at which kinesin spends a substantial fraction of its step under realistic experimental conditions. Consequently, that is the most common nucleotide state which would be under the influence of electric pulses. To that end, we analyzed the current knowledge about kinesin-1 which we used here and the consensus model for its chemo-mechanical cycle^47^. The kinesin motor domains bound to tubulin spend a substantial fraction of the step in ADP or ADP(P) state, i.e. the state when there is either ADP or ADP with a hydrolyzed phosphate group (see states 1, 2, 3, 4, and 6 in^47^, Fig. 5 therein). Therefore we decided to reproduce a situation where the kinesin binds ADP. It was also demonstrated that ADP state has a lower contact surface area with tubulin compared to ATP and APO state^46^ (Fig. S4 therein) which is corroborated by experimental observations that ADP kinesin requires a lower external force to be detached from tubulin than the ATP state^48^. These facts make the kinesin in ADP state an optimal target for EF perturbation. The final structure before the simulation is depicted in Fig. 1b,c.

We analyzed five conditions of EF exposure. No EF (control simulation) and 100 MV/m with the EF vector in directions X, −X, Y, and −Y. The simulated structure did not include the tubulins from the neighbor protofilaments which might have affected the kinesin-tubulin energetics if we applied the EF in Z and -Z directions and hence we excluded these directions from the simulation.

### Electric field affects total kinesin dipole moment magnitude

**Figure 2:**
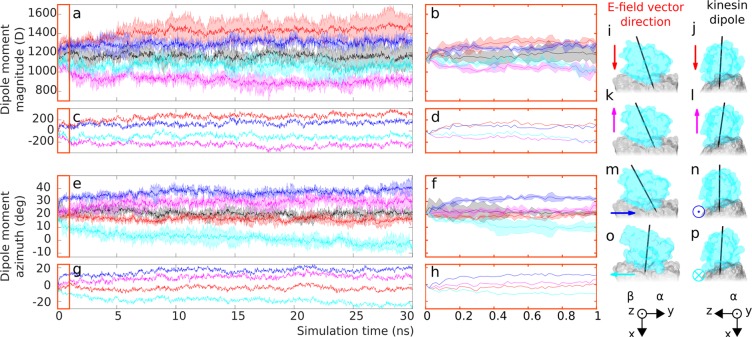
Plot of kinesin dipole moment dynamics, for five different electric field conditions: no electric field applied (black), 100 MV/m X direction (red); −X direction (magenta), Y (blue), −Y (cyan). The electric field is applied throughout the whole duration of the simulation. The thick line represents the average value and the shaded error bar depicts standard deviation (from N = 3 simulation replicates). (**a**,**b**) Display dipole moment (DM) magnitude for the whole duration of the simulation and for the first 1 ns, respectively, (**c**,**d**) display the differences of the average (across three simulation repetitions) DM magnitude values at individual field directions compared to no field condition. (**e**,**f**) Display dipole moment azimuth for the whole duration of the simulation and for the first 1 ns, respectively, (**g**,**h**) display the differences of the average values at individual field directions compared to no field condition. Images of kinesin bound to tubulin (**i**–**p**) from the end of the simulation, the kinesin dipole moment is depicted by the black arrow and the electric field vector *E* by the arrow color coded consistently with the data lines color.

Obvious feature to be analyzed when dissecting the effects EF on the kinesin is its overall dipole moment (DM) magnitude. DM magnitude is one of the basic protein electric features which, in contrast to the protein net structural charge, can be influenced by EF. In Fig. 2a, we see that the kinesin DM magnitude is fluctuating roughly around 1,200 debye for no EF condition (black line). When the 100 MV/m EF is applied along X direction (red line), the DM magnitude increases by ca. 150 debye within 1 ns (Fig. 2b,d) and then tends to increase further reaching 1,500 debye by end of the simulation (Fig. 2a). This can be understood: the EF in the X direction is polarizing the kinesin by pulling it from tubulin (yet the kinesin is bound to tubulin). The X direction of the EF is also very close to kinesin dipole orientation (Fig. 2i,j) so that any kinesin elongation deformation along the X-axis will tend to increase the DM magnitude. Slightly larger variation (error bar) for the X EF is partially caused by larger differences in the system evolution between individual trajectories compared to other field directions (Fig. S1).

The EF in −X direction Fig. 2 is causing a decrease of DM magnitude down to 900 debye (Fig. 2a - magenta line). This can be understood by electric forces acting in an opposite direction compared to that of X direction (Fig. 2k,l): the negatively and positively charged kinesin segments are being pushed towards and from the tubulin, respectively, hence decreasing the kinesin DM magnitude.

When the kinesin-tubulin system is exposed to Y and −Y EF direction (Fig. 2a,b - blue and cyan line respectively), the changes of kinesin DM magnitude are weaker than those due to X and −X EF direction. The Y and −Y EF directions increase and decrease kinesin DM magnitude by ca. 200 and 100 debye (Fig. 2c), respectively, compared to that of no EF condition.

### Electric field affects kinesin dipole moment orientation

Additionally to understanding of the EF effects on the overall DM magnitude, we also analyzed effects on the dipole moment orientation. We plot the time evolution of the dipole moment azimuth angle in Fig. 2e–h for all conditions considered. See Fig. S14 for the definition of azimuth angle. When no EF is applied, the kinesin dipole moment undergoes fluctuations due to thermal motion (see Fig. 2e,f - black line). Under such conditions, the azimuth angle of the kinesin dipole moment has an average value of 20.9 ± 1.8 degrees. However, when the 100 MV/m EF is applied, the dipole moment angle is affected. The dipole moment of the kinesin tends to get aligned with the EF vector (see for instance Fig. 2m,o) - this effect determines the change of the dipole moment vector azimuth. For the X EF direction, this effect is the weakest (Fig. 2e–j - red line) from all conditions where the EF was applied: only ca. −5 degrees shift occurs by end of the simulation (Fig. 2g - red line). The −X EF direction displays a somewhat stronger effect on the dipole angle. We see in Fig. 2h (magenta line) that ca. 5 degree shift occurs already within the first 1 ns. Beyond this time, there is a continuing trend of the angle change (Fig. 2e - magenta line). The average DM azimuth in the last 5 ns of the simulation is 15.5 ± 1.2 and 29.7 ± 1.7 degrees for X and −X EF direction, respectively.

When the EF is applied in Y and −Y direction, the angle displays a biphasic time behavior (Fig. 2e), qualitatively similar to that of DM magnitude. While the magnitude of change is similar, the time scale is slightly different. There is an abrupt change of angle by ca. 10 degrees within 200 ps of the simulation (Fig. 2f,h - blue and cyan line). After that moment, there is a continuous trend shifting the value of angle up to 39 ± 1.6 degrees (average from the last 5 ns) for Y EF orientation (Fig. 2e,g - blue line) and even by more than 20 degrees (Fig. 2g - cyan line) down to −3.2 ± 1.3 degrees for the −Y EF direction (Fig. 2e - cyan line).

Summarizing the data from the analysis of DM magnitude and its angle, we see that X and −X EF directions have a more pronounced effect on the DM magnitude (Fig. 2a–d, red and magenta line) and the Y and −Y EF directions on the DM angular orientation (Fig. 2e–h - blue and cyan line). This effect is understandable since the torque on the dipole in the EF is higher when the EF and dipole vectors are mutually perpendicular (Y and −Y EF direction - Fig. 2m–p) than when they are almost parallel (X and −X EF direction - Fig. 2i–l).

While we see a substantial rotation of kinesin dipole moment angle by ±20, we see only minor changes in overall kinesin orientation in all EF conditions (Fig. S4) compared to no EF condition (Fig. S3). This substantial dipole rotation while negligible overall rotation of the kinesin could be explained by a localized deformation/shift of charged residues. We show in the following that localized displacements of kinesin segments which contain charged residues (see their list in Supplementary information S1, section *Kinesin charge analysis*) indeed take place.

### Atom displacement analysis

**Figure 3:**
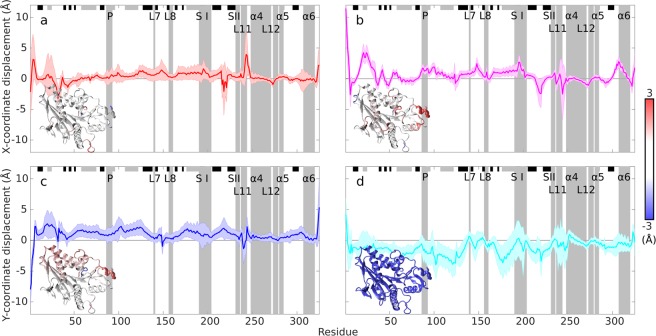
Displacement analysis of kinesin C_*α*_ atoms for 100 MV/m electric field from the last 5 ns of the simulation for the field vector in the direction (**a**) X, (**b**) −X, (**c**) Y, (−Y). For each direction, we show the difference of x (X and −X field direction) or y (Y and −Y field direction) component of the C_*α*_ atom coordinate between field and no-field condition. Mean (thick line) and standard deviation (shaded error-bar envelope) is calculated from N = 750 frames (last 5 ns (250 frames), N = 3 simulations at each field direction). Important kinesin segments are highlighted by grey bars: nucleotide binding pocket containing P-loop (P), switch I (S I), switch II (S II), and MT-binding motifs loop L7, loop L8, loop L11, *α*4 helix, loop L12, *α*5 helix, and *α*6 helix. *α*-helices and *β*-sheets are denoted by grey and black horizontal bars on the top, respectively. Kinesin molecular model shows color-coded displacement (range from −3 to +3 Å) mapped on the protein secondary structure.

In order to obtain an atom-level understanding of the EF effect on kinesin, we analyze the atomic displacement of the kinesin structure relative to no EF condition. We decided to perform an analysis of the displacement of each kinesin residue (represented by C_*α*_ atoms). The results are presented in Fig. 3. Both the magnitude of displacement (given by the mean - thick line) and the standard deviation of displacement (given by the shaded error bars) give useful information. It is often a flexible kinesin segment containing charged residues in unstructured loops which demonstrate substantial displacement, such as unstructured loops (residues 15–35, 215–225, and 235–250 in Fig. 3a,b). Within functionally relevant kinesin segments, we can see that a substantial displacement is mainly in the switch I (nucleotide binding site) and L11 segment (part of a microtubule binding motif) under all EF directions. −Y EF direction (Fig. 3d) causes a substantial displacement of a large fraction of the whole kinesin. Additionally, −X and −Y EF directions also cause observable displacement of P-loop (nucleotide binding site) and −X furthermore in *α* 6 helix (microtubule binding motif). There is also a substantial difference in the variation (depicted by error bars in Fig. 3) of the displacements: while −X EF direction manifests a minimal variance, the variance due to X, Y EF direction is higher and for −Y the highest. A smaller variation of displacement for −X than for X EF direction is also supported by principal component analysis of the kinesin motion (Fig. S6 and S7): while there is only a single cluster for the EF in −X direction (Fig. S10), there is a larger spread of data points to two clusters for the X direction of EF (Fig. S9). The slightly higher variation of displacement for −Y than Y EF direction seen in Fig. 3c,d is also supported by principal component analysis results: slightly larger spread is visible for −Y data (Fig. S12) than for Y EF direction data (Fig. S11).

These findings could be interpreted via straightforward electrostatic arguments: for the −X EF direction, the kinesin, due to its negative charge, is pushed against the tubulin, so its motion tends to be more restricted. In contrast to that, X EF direction tends to pull kinesin from the tubulin. For EF in −Y and Y directions, additionally to the pull parallel to microtubule body, also exerts a torque on the kinesin via its dipole moment.

### Atom fluctuations analysis

**Figure 4:**
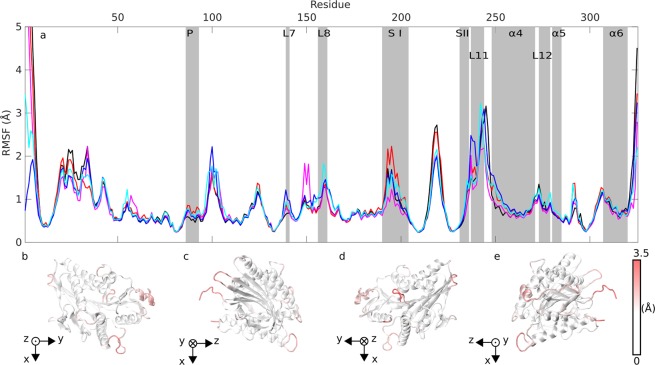
Root mean square fluctuations (RMSF) of C_*α*_ atoms for 100 MV/m electric field from 10 to 30 ns: (**a**) no field (black), X (red), −X (magenta), Y (blue), −Y (cyan) direction of the field vector. Functionally important kinesin segments are highlighted by grey vertical bars: nucleotide binding pocket containing P-loop (P), switch I (S I), switch II (S II) and microtubule-binding motifs loop L7, loop L8, loop L11, *α*4 helix, loop L12, *α*5 helix, and *α*6 helix. (**b**–**e**) RMSF of amplitude mapped on the kinesin structure for no field applied, four different viewing angles.

To understand how EF affects the dynamics of the kinesin behavior, we performed root mean square fluctuation (RMSF) on each *α* carbon (C_*α*_) to represent each residue. The results are depicted in Fig. 4 for each EF condition. We focused on functional kinesin segments (highlighted by grey vertical bars) and asked the question: do we observe a different behavior of these segments under EF exposure from that when the EF is absent? We see that segments of the nucleotide binding pocket are affected: P-loop displays slightly higher fluctuations under EF exposure (Fig. 4) compared to no EF. Switch I, known to be a flexible part of kinesin^46^, undergoes higher fluctuations for the X EF direction and lower fluctuations for −X EF direction. This opposite behavior of fluctuations in X vs. −X EF directions is corroborated by the data in Fig. 3a,b, where the shaded error bar of displacement, which correlates with fluctuation magnitude, is larger at the switch I for X EF than for the −X EF direction. We may speculate that affecting the fluctuation of the nucleotide binding site could affect the rate of ATP hydrolysis or the rate of ADP exchange - hence hypothetically affecting the kinesin stepping.

Furthermore, also the fluctuations related to few segments belonging to the microtubule binding motifs are affected by EF: loop 8 for −Y EF direction and the first half of the loop 11 for Y EF direction shows the largest difference (an increase of fluctuations) compared to no EF. Furthermore, the fluctuations of neighboring *α*4 helix is changed when exposed to EF: among all EF directions, the Y EF direction tends to increase fluctuations, especially of the first part, of *α* 4 helix most strongly. We suggest that EF-induced increase of fluctuations in these motifs might change the kinesin-tubulin interaction hence potentially destabilizing the binding of kinesin motor domain to its track.

### Energetics of kinesin-electric field interaction

The fact that we observe EF effects on the kinesin structure and dipole moment provides us also insights into kinesin-tubulin interaction energetics. We see a fundamental electrostatic interactions playing an important role here. The kinesin dipole moment is pointing towards tubulin body (Fig. 2i–p), which has an overall negative charge^20^, as would be predicted by simple electrostatics. However, the kinesin net charge is also negative (−5 e for pH 7 - see Supplementary information S1, section *Kinesin charge analysis*), which would suggest repulsive forces from tubulin, pointing to an intricate complexity of the kinesin-tubulin electrostatic interaction.

Overall charge and dipole characteristics of kinesin can help to decipher the mechanism of action of the EF on kinesin. The EF ***E*** acts on kinesin motor domain by linear (electrostatic) force ***F***_***E***_ by acting on its charge *q* (***F***_***E***_ = *q* · ***E***). For 100 MV/m EF strength we used in our simulations, ***F***_***E***_ has magnitude of ca. 0.8 pN. This value is on the lower range of forces required to rupture non-covalent bonds (pN–nN)^49^ and close to the force range required to detach ADP kinesin from microtubule (1–4 pN, acting however over time scales of ms)^48,50^. The EF also acts by torque on the kinesin dipole. The interaction energy of kinesin dipole and applied EF is given by *U* = ***p*** · ***E***, where ***p*** is the kinesin dipole moment vector and ***E*** the EF vector. For *E* = 100 MV/m and *p* = 1,150 debye, the interaction energy exceeds 90 kT (at room temperature 296 K) which explains acute effects of the EF on kinesin angle in Fig. 2e–h. This value of the interaction energy is also significantly higher than the free energy balance of the whole chemomechanical cycle of kinesin (−21.6 kT)^51^ (Chapter 1 therein) which generally suggests potential effects of EF on kinesin stepping choreography.

### Contact surface area between kinesin and tubulin

**Figure 5:**
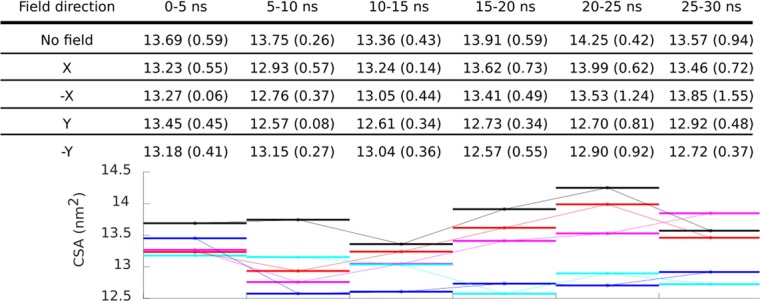
Contact surface area between kinesin and tubulin. Average (standard deviation) for each 5 ns long time interval is from N = 750 data points (250 data points from three MD run replicates). The graph displays averages for each 5 ns internal, the connecting line is plotted to guide reader’s eye. The color coding is consistent with earlier figures: black (no field), red (X EF direction), magenta (−X EF direction), blue (Y EF direction), and cyan (−Y EF direction).

Contact surface area (CSA) between kinesin and tubulin is a quantitative measure of the stability of the binding interface between kinesin and tubulin^46^. Since results we obtained in the analysis above suggest that EF might affect the segments of kinesin responsible for binding to tubulin, we decided to analyze the CSA for all field conditions. The results are in Fig. 5. The CSA in the first 5 ns is around 13.7 nm^2^ for no field condition and slightly lower for conditions with EF. The standard deviations are rather large and overlapping as there are substantial variations among individual trajectories (see Figs. S15–S19). Following the trend of the average values (Fig. 5): the EF tends to decrease the CSA for all conditions in the first 15 ns. However, after 15 ns, the effect on the CSA seems to endure substantially only for Y and −Y EF direction lowering CSA to 12.7 nm^2^. These findings corroborate the results from our dipole moment azimuth analysis: the Y and −Y EF direction is more effective in rotating the dipole moment. Rotation of the dipole moment is related to displacement of flexible charged residues, see Fig. 3 and Table S1. Such displacement is part of torque action of the electric field on the kinesin dipole and is accompanied by decreased kinesin-tubulin CSA. Thus, especially Y and −Y field directions might have a destabilizing effect on the kinesin-tubulin binding.

### Limitations, future work, self-consistency, and experimental verification

Here we discuss limitations of our current results which unravel directions for the future work. In principle, there is always a possibility to perform calculations on a longer time scale, on larger molecular systems or with higher physical accuracy. The time scale of our current simulations was 30 ns (three replicates for each condition), and the full atom molecular system was a kinesin motor domain docked on single tubulin - analyses of comparable times scale and molecular size are common in this field^20–22^. While we consider these settings to be appropriate for the first solid assessment of ns electric field effect on kinesin nanomotor, we are aware of several avenues for future work. In general, there are two strategies for enhancing molecular simulations. On the one hand, one is to increase quality of the physical approximations by including electronic polarizability in the force field^52^, quantum effects^53,54^ or to employ a quantum mechanical simulations^55^. While such approaches can grasp reality with higher accuracy, including the changes in the chemical bonds, the computational requirements increase tremendously, so only smaller molecular systems on very short time scale are tractable. A potential avenue for further research along this direction would be to assess the effect of intense electric field on ATP hydrolysis site. On the other hand, one may attempt to compute the behavior of larger systems or on larger times scales, potentially by coarse-graining the molecular system, which reduces computational demands^56,57^. Important progress in this direction already enabled the modeling of the whole (ms time scale) kinesin step^58^, which might be relevant for the assessing the effects of much weaker and longer (s and ms) electric pulses. In the current work, we selected 100 MV/m field strength since the similar field strengths have been used in earlier related works^20,21^. Furthermore, the interaction energy of the kinesin dipole and this electric field significantly exceeds kT, see section *Energetics of kinesin-electric field interaction*, so that any effect has a chance to be manifested rapidly - at the nanosecond time scale. The limitation one can argue about is that the field strength value of 100 MV/m we used in our simulations, may not be experimentally attainable for a sufficiently long time (few nanoseconds) before the dielectric breakdown of the system occurs. However, MD simulations approach is typically suitable to provide a relative assessment of various effects and may not necessarily give absolute values which could be translated to the experimental settings directly. For instance, it was found that the binding free energies of specific protein ligands predicted from MD simulations may differ two-fold or more from experimental values^59^.

Future work with larger molecular systems includes kinesin motor domain with several tubulin heterodimers arranged to reproduce a microtubule, lattice for more accurate modeling of the motor environment. There, not only the electric field direction should be varied, but also the effects due to various field strength should be analyzed.

Although not necessarily providing predictions highly accurately in their quantitative aspects, the molecular dynamics simulation can be still a powerful method to guide the experiments. For instance, the angular shift of the kinesin dipole moment for Y and −Y electric field directions (Fig. 2e,g,m–p) might suggest a destabilizing effect on kinesin-tubulin interaction potentially leading to a detachment of kinesin motor domain promoting either back-step (for Y direction) or detachment of the whole kinesin nanomotor from the microtubule track. One way how to assess the validity of predictions from MD simulations is the consistency among the results. The more substantial effect on dipole moment angle by Y and −Y than by X and −X EF direction is consistent from the perspective of electrostatic laws: larger torque on the dipole when EF vector is orthogonal to dipole moment vector. Furthermore, we see a stronger effect of Y and −Y than for X and −X EF direction on the kinesin-tubulin contact surface area, which is again consistent with a higher torque acting on kinesin. Therefore, there seems to be an overall self-consistency of the results supporting their validity.

The ultimate way to verify predictions from MD is to test them experimentally. One possible qualitative outcome suggested by our results is the effect on the kinesin stepping choreography and potentially kinesin head detachment from the microtubule. To test these predictions, one should ideally employ *in vitro* reconstituted systems of stabilized microtubules and with active kinesin motors^60^. To deliver nanosecond electric pulses to such systems, an appropriate experimental setup^61^, ideally on-chip^62^ should be integrated to the microscope. To observe the effects on kinesin, single-molecule imaging techniques such as total reflection fluorescence microscopy^63^ or interferometric scattering microscopy^64^ should be used.

## Conclusion

For the first time, we analyzed via molecular dynamics simulations how intense nanosecond-scale EF affects the structure and dipolar properties of the kinesin nanomotor. To that end, we have identified a proper conformation of the kinesin motor domain docked on the basic building block of its track, tubulin heterodimer. Next we set up molecular dynamics simulations with no EF applied as a control and 100 MV/m EF applied towards the minus and plus end of the microtubule track (Y and −Y directions, respectively) as well as parallel and antiparallel to the dipole moment of the kinesin motor (X and −X directions, respectively). We analyzed the results from molecular dynamics simulations and found that the strongest effect on the dipole moment magnitude of kinesin is for the EF in the X and −X direction. However, rotation of the kinesin dipole moment, given by the change of the dipole moment azimuth angle, is largest for the Y and −Y EF directions. Furthermore, we found that EF causes a displacement of kinesin and affects fluctuations of the kinesin segments responsible for the ATP hydrolysis as well as for the interaction of the motor with its track. We also demonstrated that Y and −Y EF directions also tend to lower the kinesin-tubulin contact surface area. Altogether, these findings suggest that EF could modify the stepping function of kinesin nanomotors. Hence, our results represent a theoretical foundation for nanosecond electric pulse-based control of kinesin nanomotors and generally contribute to the development of novel electromagnetic methods for nanobiotechnology. Our results might have a potential impact also beyond nanotechnology. Findings we delivered here also bring insights into mechanism of biological effects of nanosecond-scale intense electric pulses, which are being experimentally explored for their biomedical applications^65^ with potential neurostimulation^66^ and therapeutic endpoints^61^.

## Methods

### Structure

Initial structures for the molecular dynamics simulation was kinesin in ADP state bound to tubulin heterodimer flexibly fitted to EM-cryo map. The structure was kindly provided to us by Srirupa Chakraborty^46^ - see the paper for detailed information of structure and molecular dynamic flexible fitting to EM-cryo map. In our figures containing residue indexes on the axis, we also highlight key structural motifs of kinesin following the labeling from^46^ (Fig. S6 and Table S5 therein), see color-coding in our Fig. 1c: nucleotide binding pocket containing P-loop (blue, Q86-H93), switch I (SI, red, R190-S204), switch II (SII, dark green, D231-E236) and MT-binding motifs loop L7 (purple, L139-K141), loop L8 (brown, H156-R161), loop L11 (yellow, K237-E244), *α*4 helix (light green, L248-G271), loop L12 (pink, T273-D279), *α*5 helix (cyan, S280-I285) and *α*6 helix (orange, Y307-Q320).

### Molecular dynamics

We prepared our molecular system in GROMACS-5.1.1 software by putting initial kinesin-tubulin protein structure in the center of truncated octahedron box with the minimal distance between the solute and the box boundary set to 13 Å. This setting generated a box with length vectors (a, b, c): 134.756, 134.756, and 134.756 Å, and angles between the box vectors (bc, ac, ab): 70.53, 109.47, 70.53. Thereafter, 55,274 TIP3P water molecules^67^, 167 K^+^ and 113 Cl^−^ atoms were put into the box with volume of 1,883.76 nm^3^ to solvate the protein structure. After energy minimization (steepest descent algorithm) and pre-equilibration part of 10 ps in constant volume and constant temperature 150 K (Berendsen thermostat), we started the equilibration part by pressure coupling with Parrinello-Rahman barostat^68^ and increasing the temperature to 300 K which was held constant by stochastic velocity re-scaling algorithm^69^. The position of all C_*α*_ atoms was restrained from motion during whole equilibration part. There was another 100 ns molecular dynamics pre-production run in constant volume and temperature (Nosé-Hoover thermostat^70^) to produce the initial ensemble for our simulations. The similar approach of coupling the molecular ensemble to thermostat only (without barostat coupling) was used also in earlier works^19,20,71^. The idea of using a thermal coupling is to take into account the dissipation of heat caused by the field^72^ while not perturbing the system by other constraints such as barostat.

In this initial 100 ns run (as in all production runs) we followed the procedure previously used by Chakraborty *et al*.^46^ and restrained C_*α*_ carbons more than 12 Å away from kinesin structure by harmonic springs with force constant of 1 kcal mol^−1^ Å^−2^ to fix the tubulin dimer structure in place to simulate fixation of the tubulin in the microtubule wall. Moreover, the length of all hydrogen-containing bonds was constrained by LINCS algorithm^73^. We run all the simulations in GROMACS-5.1.1 software^74,75^ with CHARMM36 all-atom additive protein force field^76,77^. We ran three simulations for each electric field condition (no field, X, −X, Y, −Y field direction), each simulation took 15 million of 2 fs steps with leap-frog algorithm^78^. We applied periodic boundary conditions for truncated octahedron. The cut-off distance of 10 Å was used for van der Waals and electrostatic short-range forces. Long-range electrostatic forces were treated by Particle mash Ewald method (PME)^79^.

### Analysis of the displacement

For the analysis of displacement effect of the EF on kinesin atoms (Fig. 3) we compared the position of C_*α*_ atoms during the last 5 ns of each trajectory with a reference structure. We took an average structure calculated from all three trajectories without EF (4,500 frames) as the reference structure. Each frame from last 5 ns (250 × 3 = 750 frames) from trajectories with applied EF was thereafter compared to this averaged frame. The results plotted in Fig. 3 shows the final statistics for X, −X, Y and −Y EF directions with mean value, and standard deviation as the shaded error bar from N = 750 frames for each C_*α*_.

### Analysis of the root mean square fluctuation

We made an analysis of root mean square fluctuation (RMSF) for individual C_*α*_ atoms over the trajectory from time 10 to 30 ns. We aligned all frames from trajectory before further processing with same reference frame for all trajectories evaluated. For the alignment, we used a set of fifteen low fluctuating atoms from kinesin backbone (C_*α*_ atoms of residues 13, 80, 81, 82, 115, 132, 133, 209, 210, 211, 212, 226, 227, 228, and 298). For each frame we minimized the deviation of these atoms from position of atoms in reference frame in the sense of least squares.

1 $$RMS{F}_{n}=\sqrt{\frac{1}{M}\mathop{\sum }\limits_{m=1}^{M}\,{({\overrightarrow{r}}_{nm}-\langle {\overrightarrow{r}}_{n}\rangle )}^{2}},$$

where index *m* runs over all evaluated frames (from time 10 to 30 ns). ${\overrightarrow{r}}_{nm}$ is the position of C_*α*_ atom of residue n at time m and $\langle {\overrightarrow{r}}_{n}\rangle$ is the average position of n-th C_*α*_ atom.

### Principal component analysis

First, we performed Essential Dynamics Analysis^80^ of kinesin motion along our three trajectories without the EF. As the first step, we joined the frames from three trajectories without the EF applied to get one long trajectory with 4,500 frames. For the analysis, we deselected the residues with high fluctuation during the trajectory such as loose ends and unstructured coils thus we were left with 157 C_*α*_ atoms for the analysis. Before the PCA analysis, we aligned these atoms from all the frames (at the sense of minimum least square) to reference structure (average structure from the trajectory) where we used just a subset (same as for RMSF analysis) of these atoms as a criterion for alignment. Thus we got the trajectory of 157 atoms and 4,500 frames. With such “adjusted” trajectory, the covariance matrix C (3N × 3N) was calculated. The element *C*_*ij*_ from the matrix is calculated as:

2 $${C}_{ij}=\frac{1}{M}\mathop{\sum }\limits_{t=1}^{M}\,({x}_{i}(t)-\langle {x}_{i}\rangle )({x}_{j}(t)-\langle {x}_{j}\rangle ).$$

Letter *x* with subscript stands for position coordinate (i = 1, 2, …, 3N; j = 1, 2, …, 3N; where N is the total number of atoms involved in an analysis (157)). Index t denotes time and runs over all frames. The value in angle bracket is a time average of coordinate i. In the next step, the covariance matrix was diagonalized and resulting eigenvectors and eigenvalues were sorted by eigenvalue from the highest to the lowest to get the most contributing modes as the first.

3 $${\mathbb{C}}={\mathbb{T}}\Lambda {{\mathbb{T}}}^{T}$$

${\mathbb{T}}$ is a column matrix of eigenvectors and Λ is a diagonal matrix of eigenvalues, ${{\mathbb{T}}}^{T}$ denotes the transformed matrix.

Next, we projected our trajectories along the first two eigenvectors. The trajectories with applied EF were prepared in the same fashion as the trajectory without the EF at the first step: i.e. by the joining the three trajectories followed by superimposing them to reference structure (i.e. average structure without the EF). The matrix of the projections of each time-step onto each eigenvector ${\mathbb{P}}$ is obtained by multiplying the trajectory matrix ${\mathbb{X}}$ (atoms run in rows, time in columns) by ${\mathbb{T}}$ the column matrix of eigenvectors.

4 $${\mathbb{P}}={\mathbb{X}}{\mathbb{T}}$$

### Contact surface area analysis

Contact surface area was calculate as a half of difference between the sum of solvent-accessible surface area (SASA) of separate tubulin heterodimer and separate kinesin and SASA of kinesin-tubulin complex. In all cases we utilize the algorithm of^81^ implemented in GROMACS tool package (gmx sasa). We set 0.14 nm for the solvent probe radius^46^.

## Supplementary information

Supplementary information S1

## Data Availability

All raw data (initial molecular structure and trajectories) are available under 10.5281/zenodo.2644158.
