# Supplementary material for: Molecular dynamics simulation of the nanosecond pulsed electric field effect on kinesin nanomotor
Source: Sci Rep. 2019 Dec 23;9:19721. doi: 10.1038/s41598-019-56052-3 (PMC6928163; doi:10.1038/s41598-019-56052-3)
Supplement: Supplementary file 1 — Supplementary information S1 [file 41598_2019_56052_MOESM1_ESM.pdf]

Supplementary information for ”Molecular dynamics simulation of the nanosecond pulsed electric field effect on kinesin nanomotor”

Jiří Průša[1,2] and Michal Cifra[1,*]

[1]Institute of Photonics and Electronics of the Czech Academy of Sciences, Prague, Czechia
[2]Faculty of Chemical Engineering, University of Chemistry and Technology, Prague, Czechia
[*]cifra@ufe.cz

ABSTRACT

We provide here supplementary data on the kinesin structural charge, dipole moment magnitude evolution for individual trajectories, analysis of the angle between gyration axes of tubulin and kinesin, principal component analysis of kinesin motion, and kinesin-tubulin contact surface area.

Kinesin charge analysis

We have identified charged residues in the kinesin structure since they represent sites where electric field acts by force.
45 negatively charged residues: 3 6 20 22 27 36 37 49 59 64 72 75 96 101 103 114 123 124 127 136 140 144 147 157 158 170 177 178 181 184 185 199 215 220 231 236 244 249 250 270 279 288 309 311 325
40 positively charged residues: 1 10 14 16 25 28 32 44 50 67 68 71 91 98 110 131 141 143 150 159 161 166 171 187 190 203 213 222 226 237 240 252 256 278 281 284 295 313 321 323.
Total kinesin electric charge: - 5 e.

Aminoacid	Counts
Glycine	20
Proline	8
Alanine	16
Valine	25
Leucine	22
Isoleucine	22
Methionine	8
Cystein	8
Phenilalanine	11
Tyrosine	14
Histidin	7
Lysine	25
Arginine	14
Glutamin	11
Asparagine	19
Glutamic acid	24
Aspartic acid	20
Serine	31
Threonine	20

Table S1. Count of amino acids in kinesin structure (1BG2).

Dipole moment magnitude evolution for individual trajectories

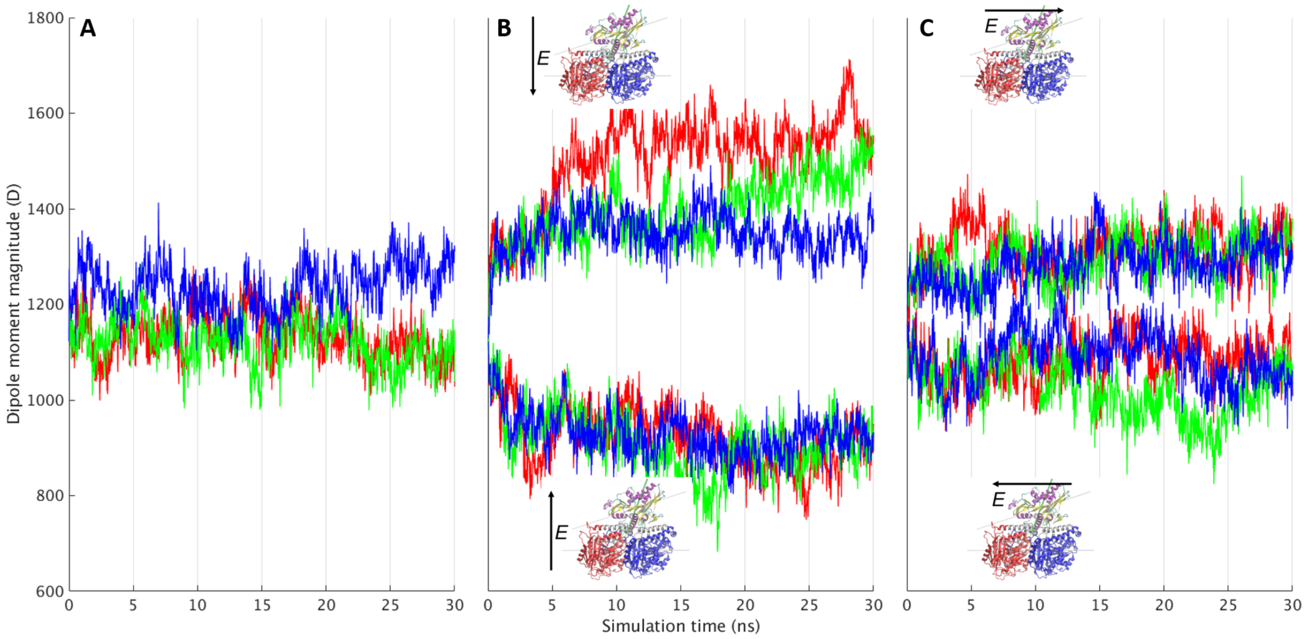

Figure S1. Time evolution of dipole moment for individual molecular dynamics trajectories, each condition N=3 trajectories. (A) no electric field, (B) X (top) and -X (bottom) field direction, (C) -Y (top) and Y (bottom) electric field direction.

Analysis of the angle between gyration axes of tubulin and kinesin

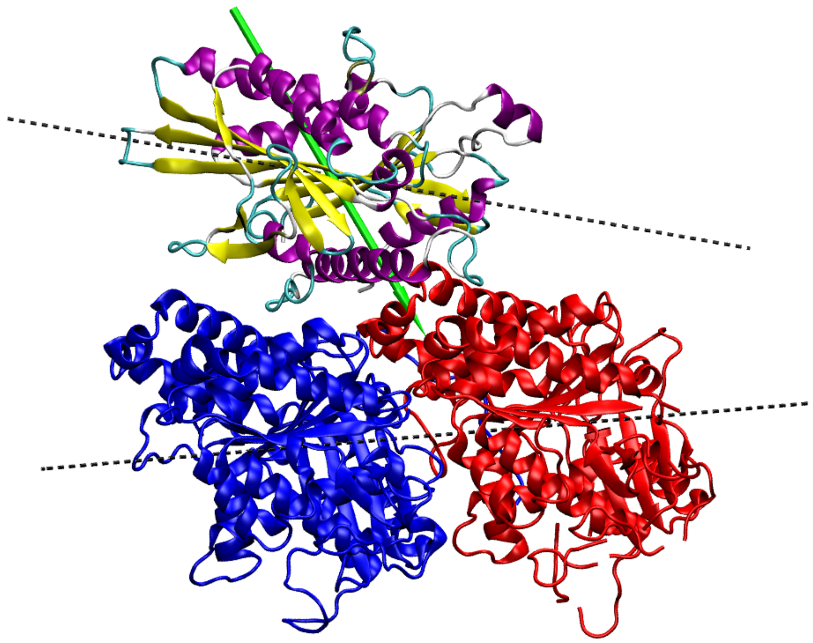

Figure S2. Our kinesin-on-tubulin structure with gyration axis depicted as dashed lines. Green arrow is the dipole moment of the kinesin.

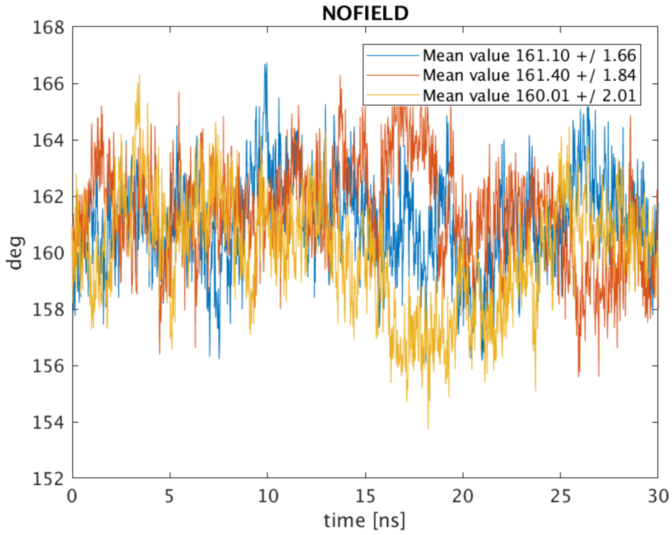

Figure S3. Time evolution of the angle between gyration axis of tubulin and kinesin for no electric field applied. N=3 simulation replicates.

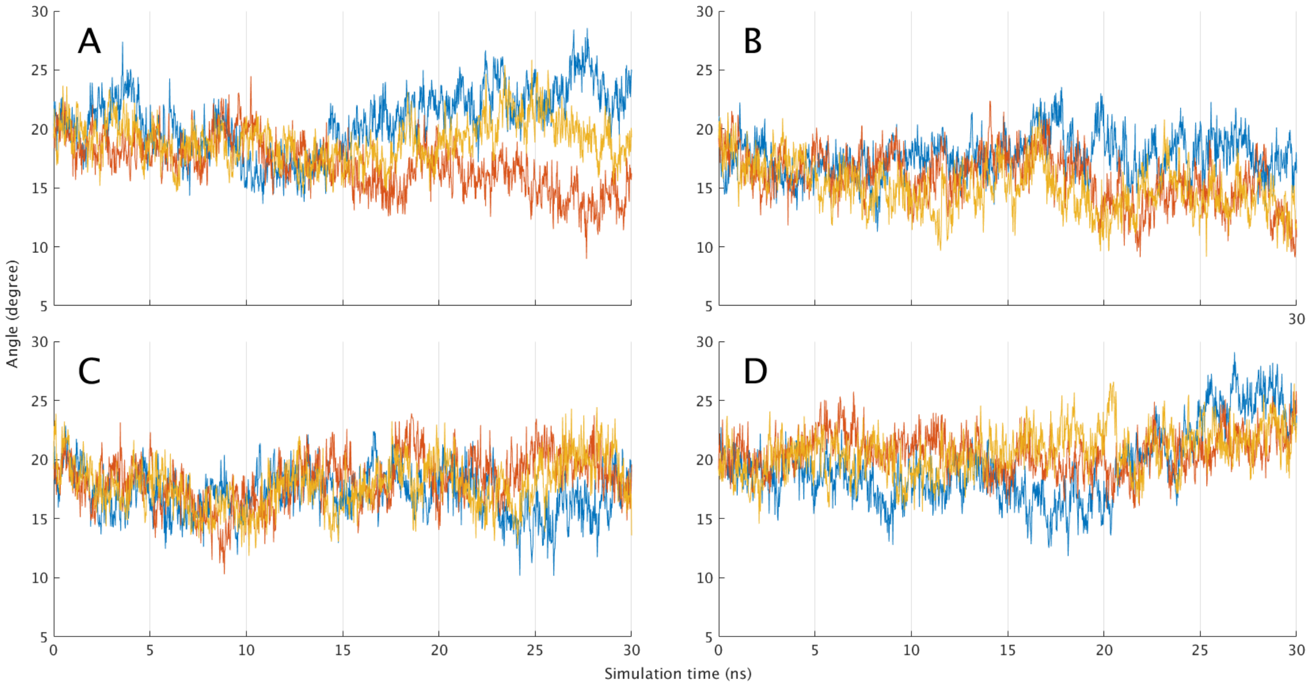

Figure S4. Time evolution of the angle between gyration axis of tubulin and kinesin. Field directions A: X, B: Y, C: -X, D: -Y. N=3 simulation replicates for each field direction.

Principal component analysis of kinesin motion

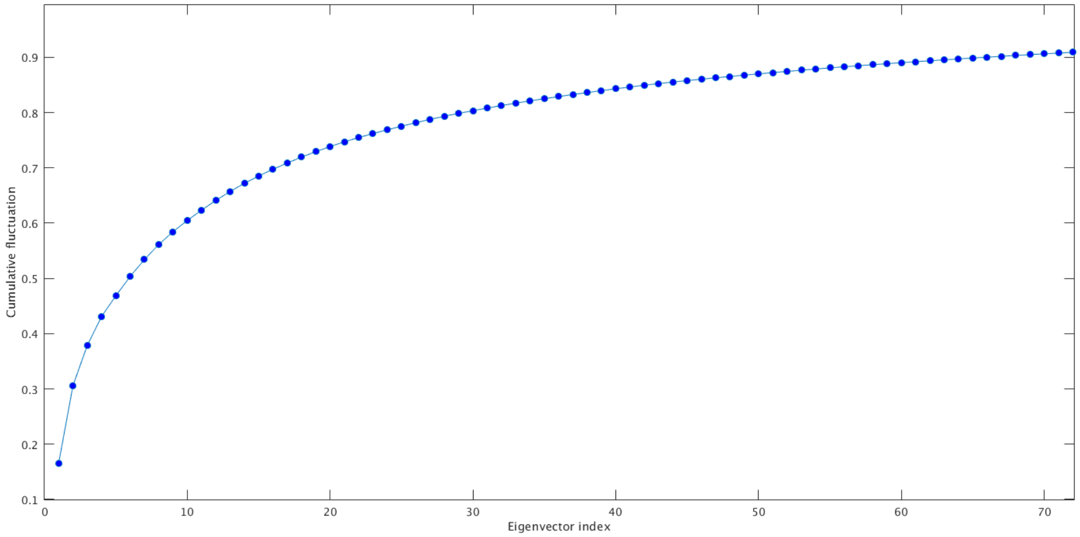

Figure S5. Cumulative fluctuations up the n-th principal components from the Principal Component Analysis demonstrate that the first two components capture 0.3 of the total kinesin motion.

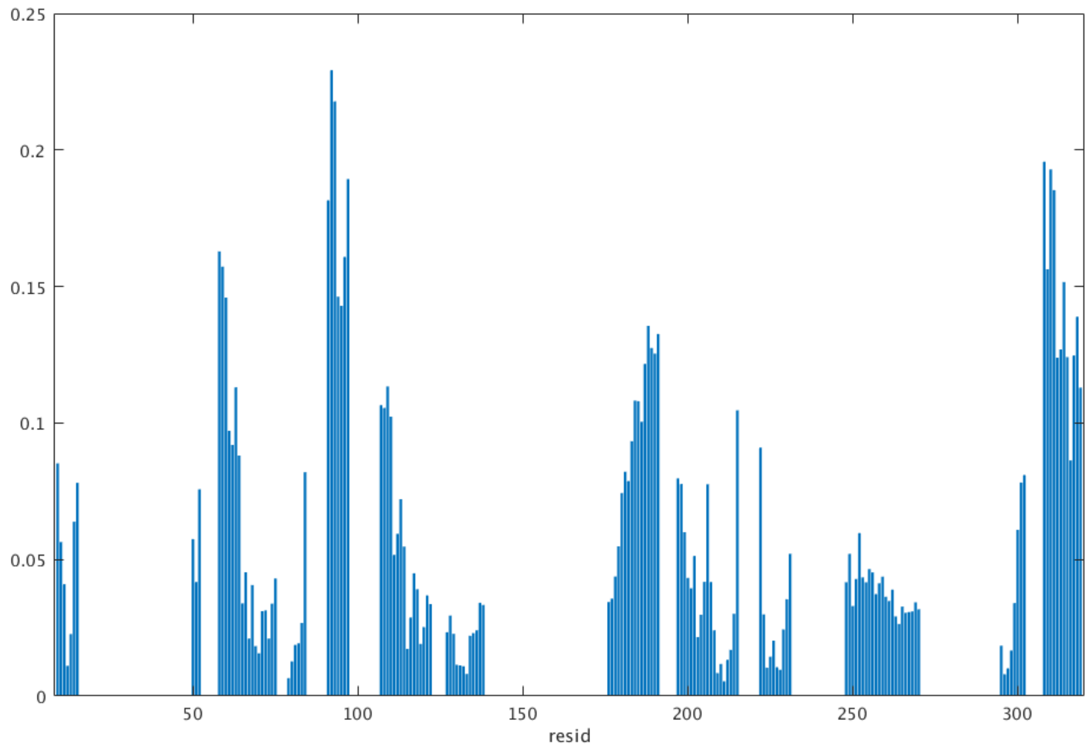

Figure S6. Residues and their weights contributing to the 1st principal component of the kinesin motion (no electric field)

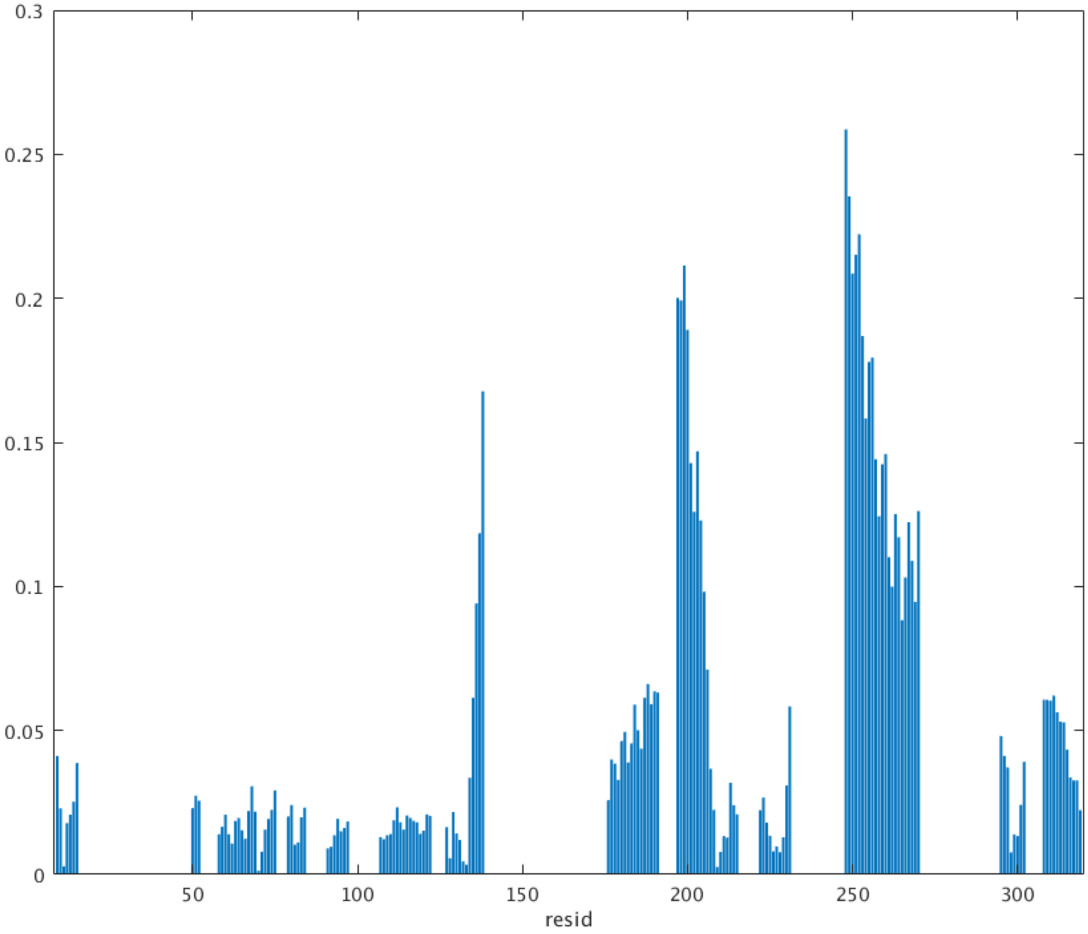

Figure S7. Residues and their weights contributing to the 2nd principal component of the kinesin motion (no electric field)

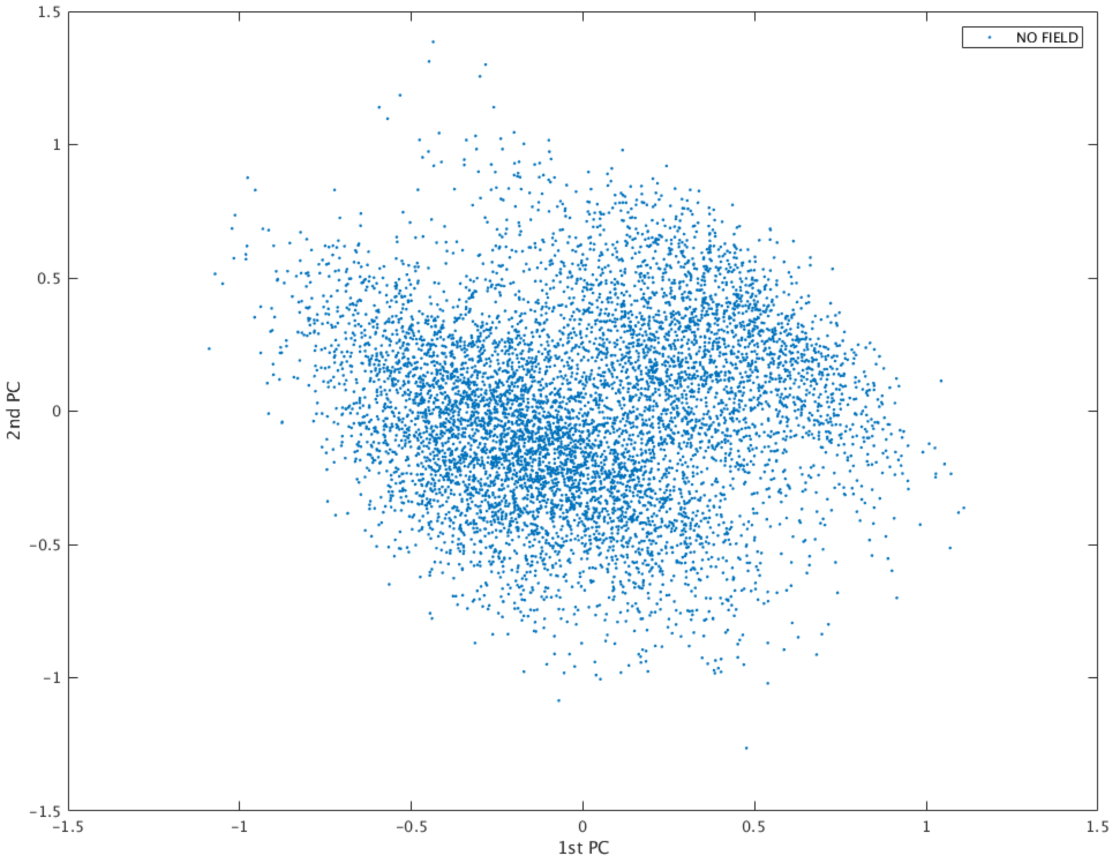

Figure S8. Clusters of kinesin motion mapped on the space of PC1 and PC2 (no electric field). Every point in the graph corresponds to one time point in the simulation. Total 4500 points from N=3 simulations are displayed.

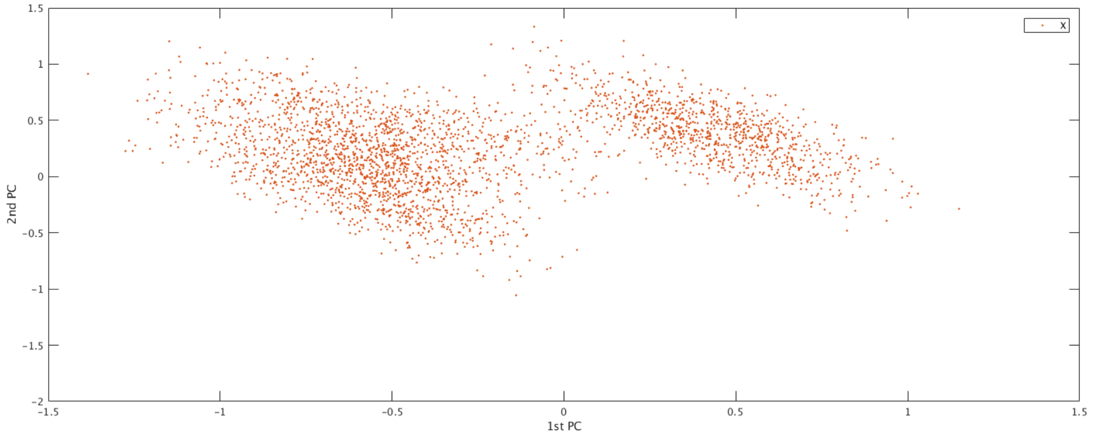

Figure S9. Clusters of kinesin motion in X direction electric field mapped on the space of PC1 and PC2 from no electric field simulation. Every point in the graph corresponds to one time point in the simulation. Total 4500 points from N=3 simulations are displayed.

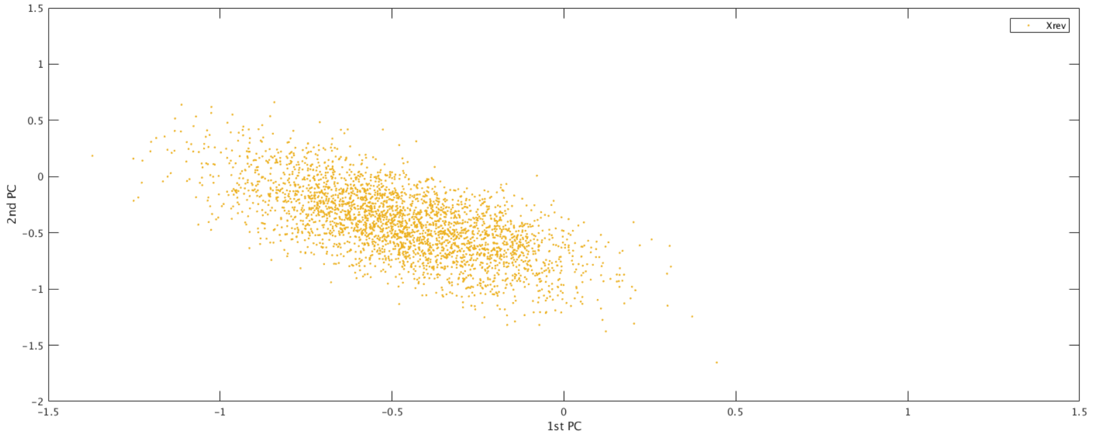

Figure S10. Clusters of kinesin motion in -X direction electric field mapped on the space of PC1 and PC2 from no electric field simulation. Every point in the graph corresponds to one time point in the simulation. Total 4500 points from N=3 simulations are displayed.

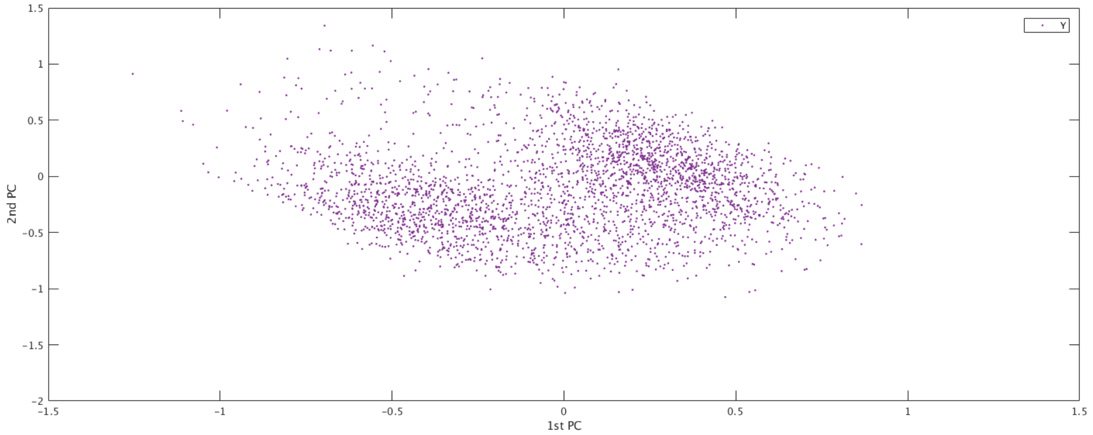

Figure S11. Clusters of kinesin motion in Y direction electric field mapped on the space of PC1 and PC2 from no electric field simulation. Every point in the graph corresponds to one time point in the simulation. Total 4500 points from N=3 simulations are displayed.

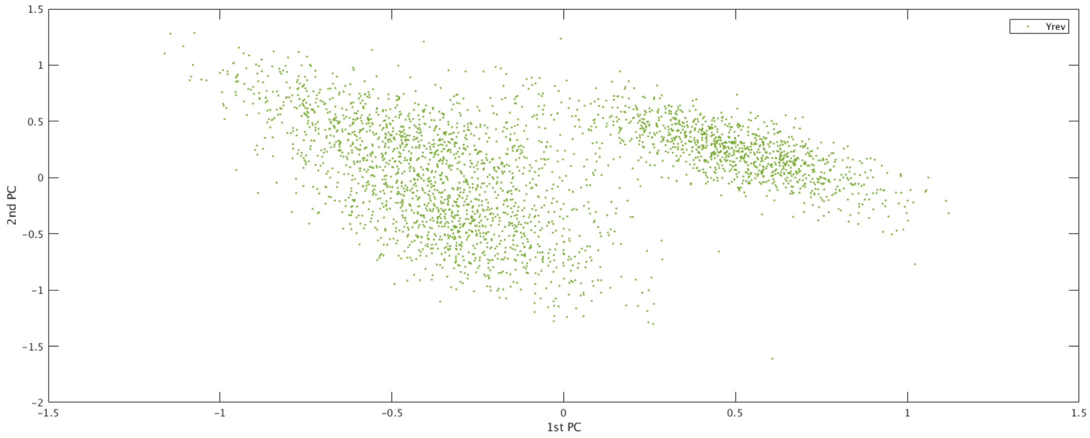

Figure S12. Clusters of kinesin motion in -Y direction electric field mapped on the space of PC1 and PC2 from no electric field simulation. Every point in the graph corresponds to one time point in the simulation. Total 4500 points from N=3 simulations are displayed.

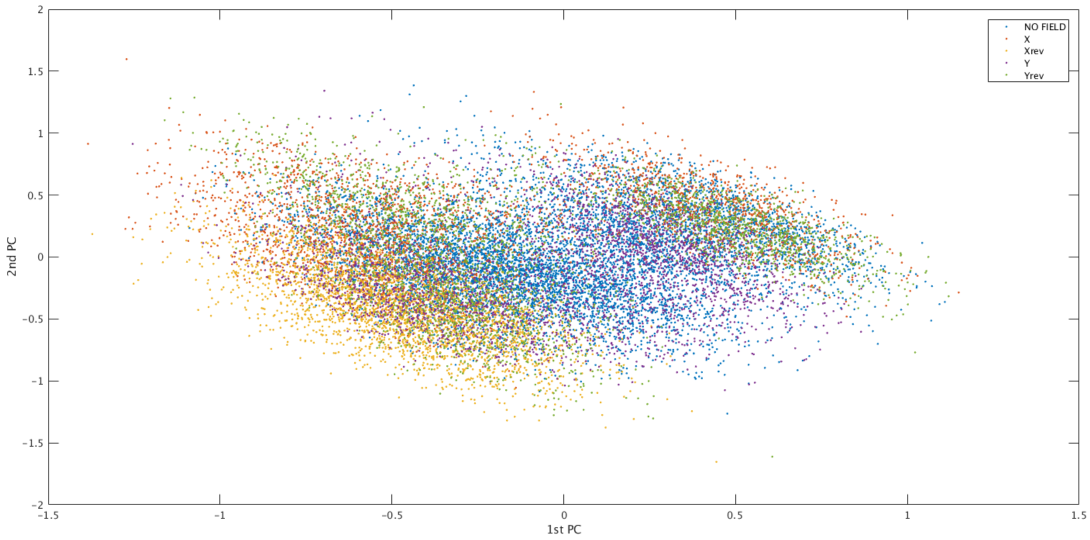

Figure S13. Clusters of kinesin motion in field conditions as well as for no field condition mapped on the space of PC1 and PC2 from no electric field simulation. Every point in the graph corresponds to one time point in the simulation. Total 4500 points from N=3 simulations are displayed for each field condition.

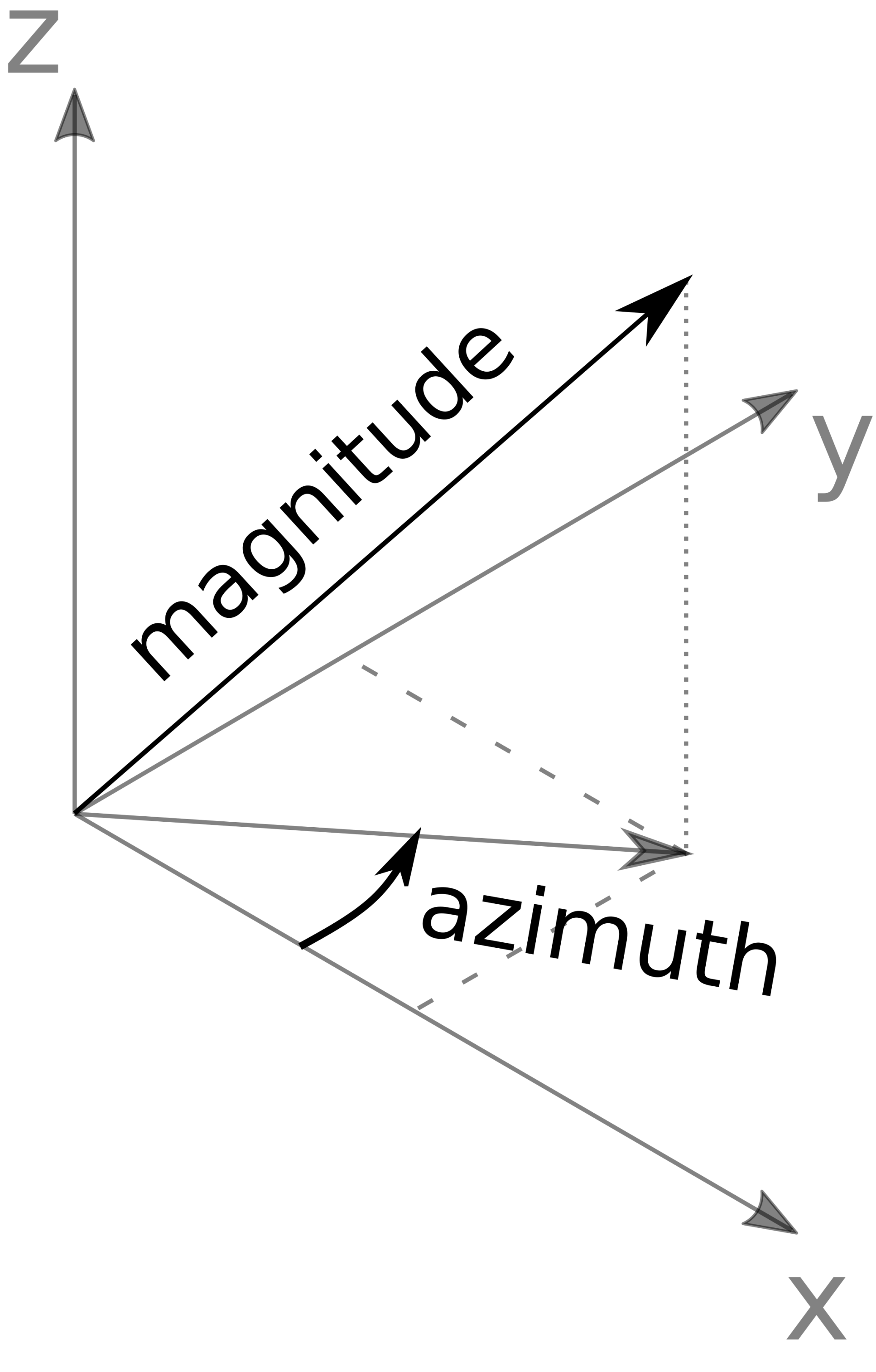

Figure S14. Mapping of three-dimensional Cartesian coordinates to spherical coordinates (elevation is missing as we do not present it out in our work). Zero azimuth corresponds to vector along x-axis in Cartesian coordinates.

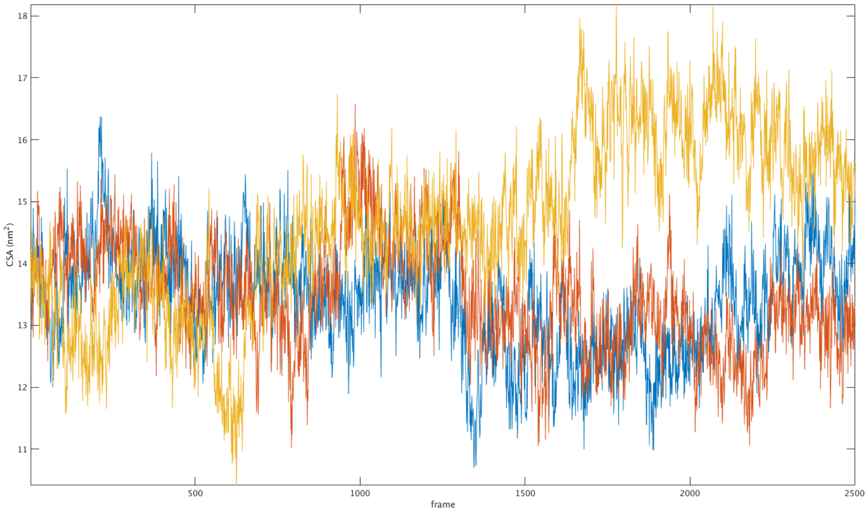

Figure S15. Contact surface area time evolution for three independent 50 ns simulations without external electric field.

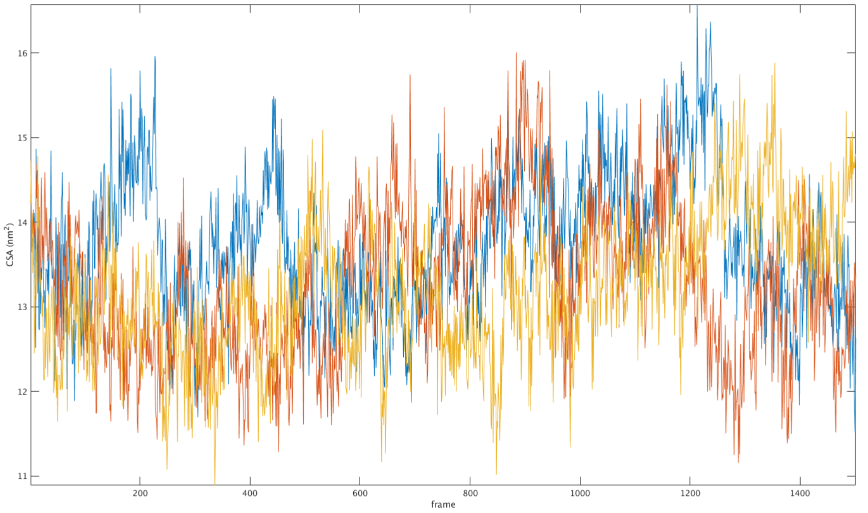

Figure S16. Contact surface area time evolution for three independent 30 ns simulations with electric field in X direction.

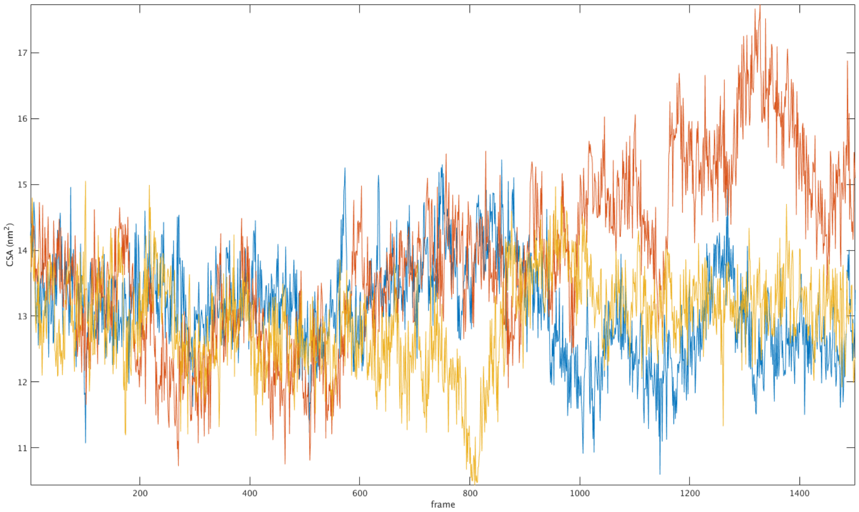

Figure S17. Contact surface area time evolution for three independent 30 ns simulations with electric field in -X direction.

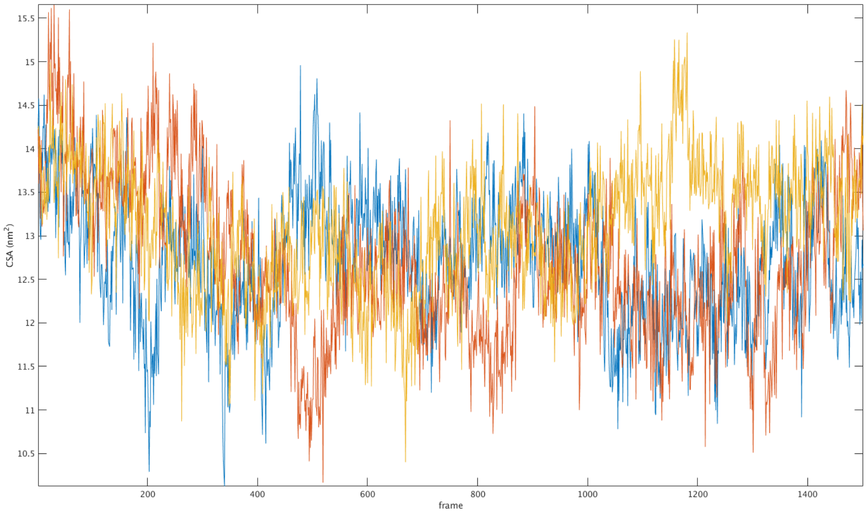

Figure S18. Contact surface area time evolution for three independent 30 ns simulations with electric field in Y direction.

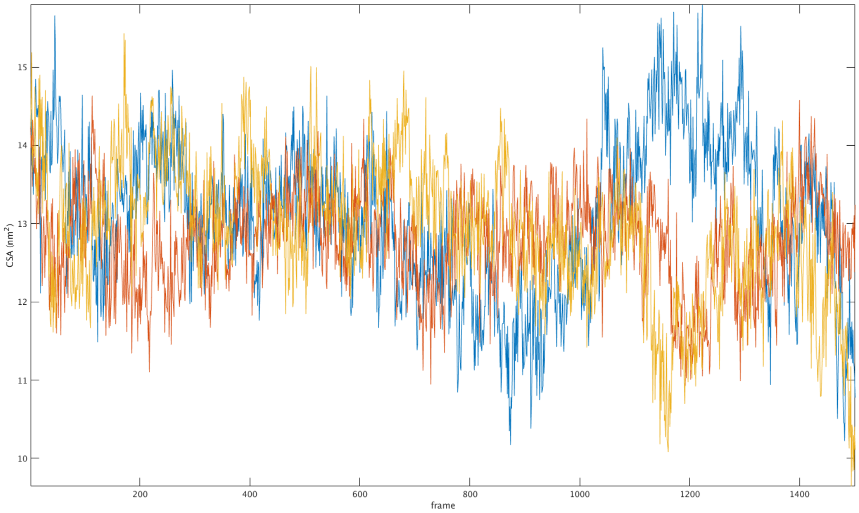

Figure S19. Contact surface area time evolution for three independent 30 ns simulations with electric field in -Y direction.
